# Obesity-associated Blunted Subcutaneous Adipose Tissue Blood Flow After Meal Improves After Bariatric Surgery

**DOI:** 10.1210/clinem/dgac191

**Published:** 2022-04-01

**Authors:** Teemu Saari, Jukka Koffert, Henri Honka, Saila Kauhanen, Mueez U-Din, Nils Wierup, Andreas Lindqvist, Leif Groop, Kirsi A Virtanen, Pirjo Nuutila

**Affiliations:** Turku PET Centre, University of Turku, 20520 Turku, Finland; Turku PET Centre, Turku University Hospital, 20520 Turku, Finland; Turku PET Centre, University of Turku, 20520 Turku, Finland; Department of Gastroenterology, Turku University Hospital, 20520 Turku, Finland; Turku PET Centre, University of Turku, 20520 Turku, Finland; Division of Digestive Surgery and Urology, Turku University Hospital, 20520 Turku, Finland; Turku PET Centre, University of Turku, 20520 Turku, Finland; Turku PET Centre, Turku University Hospital, 20520 Turku, Finland; Department of Clinical Sciences, Lund University Diabetes Centre, 20213 Malmö, Sweden; Department of Clinical Sciences, Lund University Diabetes Centre, 20213 Malmö, Sweden; Department of Clinical Sciences, Lund University Diabetes Centre, 20213 Malmö, Sweden; Turku PET Centre, University of Turku, 20520 Turku, Finland; Turku PET Centre, Turku University Hospital, 20520 Turku, Finland; Institute of Public Health and Clinical Nutrition, University of Eastern Finland, 70211 Kuopio, Finland; Turku PET Centre, University of Turku, 20520 Turku, Finland; Turku PET Centre, Turku University Hospital, 20520 Turku, Finland; Department of Endocrinology, Turku University Hospital, 20520 Turku, Finland

**Keywords:** adipose tissue, bariatric surgery, blood flow, positron emission tomography, type 2 diabetes, glucose-dependent insulinotropic polypeptide

## Abstract

**Context:**

Glucose-dependent insulinotropic peptide (GIP) and meal ingestion increase subcutaneous adipose tissue (SAT) perfusion in healthy individuals. The effects of GIP and a meal on visceral adipose tissue (VAT) perfusion are unclear.

**Objective:**

Our aim was to investigate the effects of meal and GIP on VAT and SAT perfusion in obese individuals with type 2 diabetes mellitus (T2DM) before and after bariatric surgery.

**Methods:**

We recruited 10 obese individuals with T2DM scheduled for bariatric surgery and 10 control individuals. Participants were studied under 2 stimulations: meal ingestion and GIP infusion. SAT and VAT perfusion was measured using ^15^O-H_2_O positron emission tomography–magnetic resonance imaging at 3 time points: baseline, 20 minutes, and 50 minutes after the start of stimulation. Obese individuals were studied before and after bariatric surgery.

**Results:**

Before bariatric surgery the responses of SAT perfusion to meal (*P* = .04) and GIP-infusion (*P* = .002) were blunted in the obese participants compared to controls. VAT perfusion response did not differ between obese and control individuals after a meal or GIP infusion. After bariatric surgery SAT perfusion response to a meal was similar to that of controls. SAT perfusion response to GIP administration remained lower in the operated-on than control participants. There was no change in VAT perfusion response after bariatric surgery.

**Conclusion:**

The vasodilating effects of GIP and meal are blunted in SAT but not in VAT in obese individuals with T2DM. Bariatric surgery improves the effects of a meal on SAT perfusion, but not the effects of GIP. Postprandial increase in SAT perfusion after bariatric surgery seems to be regulated in a GIP-independent manner.

The responses of adipose tissue (AT) to natural cues or challenges, such as eating, and related gastrointestinal hormones are not thoroughly understood in obesity. The normal physiological response of AT to meal ingestion is the uptake of nutrients and storing of triglycerides (TGs) in intracellular lipid droplet(s). In obesity, insulin resistance of AT is one major factor contributing to unbalanced insulin response, and furthermore in type 2 diabetes mellitus (T2DM), AT becomes a sink of glucose: Increasing fat mass can compensate for reduced glucose uptake per mass of AT (1).

Bariatric surgery is an effective way to achieve rapid and sustained weight loss (2). In addition, weight loss after bariatric surgery is related to improved glucose homeostasis, insulin sensitivity, and a healthier circulatory lipid profile. Bariatric surgery has been shown to cause remission from T2DM, prevent development of future T2DM, and reduce cardiovascular complications (3, 4).

Glucose-dependent insulinotropic peptide (GIP) is an incretin hormone produced by K cells in the proximal small intestine, and it is released in response to the ingestion of a meal containing either glucose or fat (5). Furthermore, GIP causes a glucose-dependent increase in insulin secretion (6).

GIP increases subcutaneous adipose tissue (SAT) blood flow in the presence of insulin (7). This response is blunted in obesity and partially normalized after weight loss (8, 9). GIP has been shown in animal studies to have an important role in regulating lipoprotein lipase and increasing systematic TG clearance (10). Different AT depots have distinct responses to hormonal stimulation, and they have been shown to react differently to weight loss (11, 12). Visceral adipose tissue (VAT) is a more metabolically active adipose depot and has been more strongly associated with obesity-related complications, such as T2DM, than SAT (13-18). Subsequent to bariatric surgery the volume of SAT is reduced more in comparison with VAT (19-22). However, there is still a gap in our understanding of how the tissue-specific metabolism of SAT and VAT is affected by bariatric surgery–induced weight loss, particularly under stimulatory conditions. Increased understanding of these processes is warranted and may provide physiological insights into the mechanism of AT shrinkage after bariatric surgery. The blood flow of AT depots represents the functional metabolic capacity of the AT as well as an ability of the tissue to mobilize free fatty acids in the lipolytic state (11, 23). Since adequate blood flow influences TG delivery to the adipocytes, the regulation of whole-body lipid metabolism is also dependent on AT hemodynamics (24). This study was designed to elucidate the physiological hemodynamic responses of SAT and VAT depots after meal ingestion and after the administration of exogenous GIP in obese individuals with T2DM, as well as the effects of rapid weight loss caused by bariatric surgery. Healthy control individuals were studied for comparison with the responses of obese participants. We hypothesized that weight loss after bariatric surgery would restore the metabolic capacity, in terms of blood flow, of SAT and VAT in response to meal ingestion and/or GIP infusion.

## Materials and Methods

**Figure 1.**
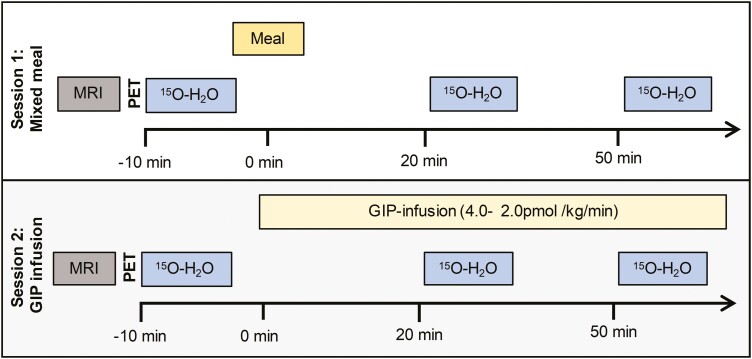
Timeline of the study protocol. All participants were scanned after an overnight fast. All participants were first scanned with magnetic resonance imaging (MRI), followed by 3 positron emission tomography (PET) scans using ^15^O-H_2_O at 3 time points: baseline, 20 minutes, and 50 minutes after stimulation by meal ingestion or glucose-dependent insulinotropic peptide (GIP) infusion. GIP infusion was started at 4 pmol/kg/min and halved after 15 minutes to mimic physiological concentrations of GIP after meal.

This is part of the GIP-PET study (ClinicalTrials.gov number NCT01880827) described previously (25-27). Briefly, 10 morbidly obese individuals scheduled for bariatric surgery (Roux-en-Y gastric bypass or vertical sleeve gastrectomy, n = 5 in each group) and 10 healthy control individuals were recruited. All study participants were nonsmokers, while all morbidly obese patients had T2DM. A washout period was designated for drugs to eliminate any effects on the study (24 hours for antihypertensives, 72 hours for antidiabetic drugs, except for long-acting glucagon-like peptide-1 [GLP-1] receptor agonists: 10 weeks). All participants underwent 2 different imaging experiments in randomized order on different days. Participants underwent imaging in the supine position with a combined positron emission tomography–magnetic resonance imaging (PET/MRI) scanner to investigate the changes in AT blood flow after a mixed meal or during GIP infusion (Fig. 1). In the mixed-meal procedure, participants ingested a 250-kcal (40 g carbohydrates, 6 g fat, 9 g protein) liquid meal (Nutridrink, Nutricia Advanced Medical Nutrition) in 10 minutes. The dose was selected to enable the ingestion of the meal in a 10-minute time frame during the scanning episodes (25). During the GIP-infusion procedure, participants received a constant infusion of GIP1-42 (Bachem Holding AG), initially at a rate of 4.0 pmol/kg/min. After 15 minutes, the rate was reduced to 2.0 pmol/kg/min with the intention to reproduce the GIP excursion seen after ingesting a mixed meal (28). Blood flow of the AT depots was measured using a ^15^O-H_2_O PET radiotracer. After a baseline ^15^O-H_2_O PET scan of the abdominal region, the scan was repeated 20 and 50 minutes after meal ingestion and in another scanning session during GIP infusion. During the scanning sessions plasma levels of glucose, insulin, GIP, and GLP-1 were measured at time points 0, 15, 30, 45, 60, and 90 minutes. The morbidly obese participants were scanned before bariatric surgery and the same protocol was repeated approximately 2 months (75 ± 25 days, mean ± SD) after surgery.

### Imaging

PET scans were performed using a Philips Ingenuity combined PET/MRI scanner (Philips Healthcare).

Tissue-specific perfusion was quantified with an intravenous injection of ^15^O-H_2_O (radiowater), and a dynamic emission scan of the abdominal region was performed. Radiowater was produced using the Hidex Radiowater Generator (Hidex Oy). Tissue-specific perfusion was calculated using the 1-tissue compartment model, a method based on the principle of exchange of inert gas between blood and tissues. Input functions were derived from the images by drawing a volume of interest in the abdominal aorta. Analysis of PET images was conducted using Carimas software (Turku PET Centre) (29, 30).

The study protocol was reviewed by the local ethical committee of the Hospital District of Southwest Finland, and the study was carried out according to the principles of the Declaration of Helsinki and Good Clinical Practice guidelines. Written informed consent was signed by all study participants before any study procedures and inclusion in the study.

### Biochemical Analyses

Plasma GLP-1 was measured by an enzyme-linked immunosorbent assay (Millipore catalog No. EZGLPHS-35K, RRID:AB 2884907) as was plasma GIP (Millipore catalog No. EZHGIP-54K, RRID:AB_2801401). Electrochemiluminescence immunoassays were used to measure plasma insulin (Roche catalog No. 12017547, RRID:AB_2756877) and C-peptide concentrations (Roche catalog No. 03184897, RRID:AB_2909476).

### Statistics

Comparison of means was performed with 2-way *t* test, analysis of variance, or Wilcoxon rank sum test. Associations between variables were calculated using the Pearson or Spearman rank sum test. Changes over time and between groups were tested with repeated-measures analyses using linear mixed models, and the Tukey-Kramer method was used to adjust the *P* values of pairwise comparisons. *P* less than .05 was considered statistically significant. Data are presented as mean ± SD. Normality of distribution was checked visually together with the Shapiro-Wilk test of normality and Q-Q plot. For changes in AT blood flow, mean difference of basal SAT blood flow 0.8 (0.4 SD) were reported by Asmar et al (8) after conventional weight loss, and using these results a sample size of 5 pairs would be needed for 80% power and 5% level of significance (2-sided). Statistical analyses were performed using IBM SPSS Statistics (version 25) or SAS JMP Pro 25.

### Calculations

Insulin sensitivity is expressed as homeostatic model assessment of insulin resistance (HOMA-IR) and 2-hour oral glucose sensitivity index (31).

AT volumes were calculated by measuring SAT and VAT from a single MRI slice at the level of L3, and calculating total SAT and VAT mass using predictive equations by Schweitzer et al (32). AT volumes were measured with a semiautomated method using Carimas software (Turku PET Centre).

## Results

General characteristics of the study individuals are provided in Table 1. The characteristics and metabolic health status of the participants have been previously reported (25, 26).

**Table 1.** Anthropometric measurements of study participants before and after bariatric surgery and control individuals

	Controls	Before surgery	After surgery	Controls vs before surgery	Controls vs after surgery	Before vs after surgery
Age, y	Mean (SD)	Mean (SD)	Mean (SD)	P	P	P
**No., male/female**	46 (9.41)	51.7 (7.01)	52.3 (6.73)	.03	.02	.005
**Height, cm**	10 (2/8)	10 (2/8)	10 (2/8)			
**Weight, kg**	165.8 (10.5)	167.7 (12.9)	167.5 (13.0)	.50	.47	.09
**BMI**	63.7 (13.7)	114.6 (18.9)	98.0 (16.3)	< .001	< .001	< .001
**Waist, cm**	23.1 (2.4)	40.8 (5.9)	35.2 (6.3)	< .001	< .001	< .001
**Hip, cm**	80.7 (10.1)	121.1 (7.6)	109.6 (9.7)	< .001	< .001	.001
**Fat, %**	95.5 (5.3)	127.3 (8.7)	115.2 (8.2)	< .001	< .001	< .001
**SAT mass, kg**	25.6 (5.9)	46.0 (9.7)	40.9 (13.5)	< .001	.001	.007
**VAT mass, kg**	14.7 (3.7)	39.7 (6.6)	32.0 (8.4)	< .001	< .001	<.001
**Plasma glucose, mmol/L**	1.6 (0.6)	6.4 (1.4)	4.9 (1.3)	< .001	< .001	<.001
**Plasma insulin, mIU/L**	5.1 (0.4)	6.5 (1.1)	5.7 (0.7)	< .001	.02	.008
**Plasma C-peptide, nmol/L**	4.5 (2.1)	19.4 (9.4)	12.2 (7.1)	< .001	.001	.03
**HOMA-IR**	0.52 (0.15)	1.22 (0.30)	1.00 (0.37)	< .001	< .001	.03
**Cholesterol, mmol/L**	1.0 (0.5)	5.9 (3.4)	3.1 (1.9)	< .001	.001	.02
**Triglycerides, mmol/L**	4.8 (1.1)	4.5 (1.4)	3.7 (0.9)	.51	.01	.01
**HDL, mmol/L**	0.8 (0.4)	1.9 (0.7)	1.1 (0.5)	< .001	.08	.005
**LDL, mmol/L**	1.9 (0.5)	1.2 (0.3)	1.3 (0.4)	.002	.005	.59
**HbA**_**1c**_ **, mmol/mol**	2.6 (0.8)	2.4 (1.1)	1.9 (0.7)	.67	.05	.06
**Age, y**	32 (3.6)	30 (4.0)	36 (2.8)	< .001	.009	.01

Data are presented as mean (SD). Unpaired Student’s t test for Control vs Before Surgery and Control vs After Surgery comparisons. Paired Student’s t test used for Before Surgery vs After Surgery comparisons.

Abbreviations: BMI, body mass index; HbA1c, glycated hemoglobin A1c; HDL, high-density lipoprotein; HOMA-IR, homeostatic model assessment of insulin resistance; LDL, low-density lipoprotein; SAT, subcutaneous adipose tissue; VAT, visceral adipose tissue.

**Figure 2.**
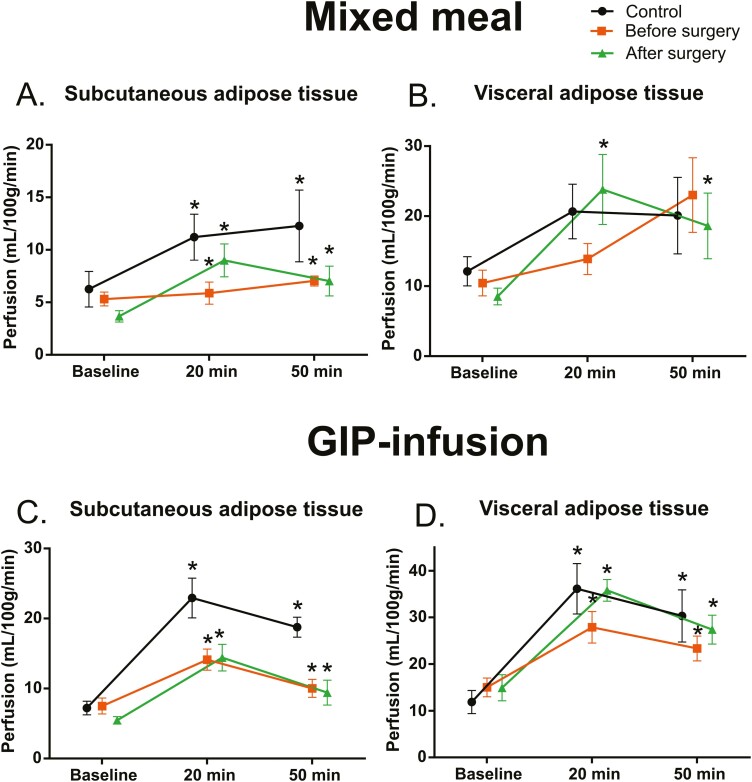
Subcutaneous adipose tissue and visceral adipose tissue blood flow at baseline, 20 minutes, and 50 minutes after a mixed meal or start of glucose-dependent insulinotropic peptide (GIP) infusion. Statistical comparison was performed using linear mixed models, and Tukey-Kramer method was used to adjust the *P* values of pairwise comparisons. Asterisk indicates a statistically significant difference from baseline (*P* < .05). Control n = 10, before surgery mixed meal n = 10, after surgery mixed meal n = 9, before surgery GIP infusion n = 9, after surgery GIP-infusion n = 9.

Baseline blood flow measurements, without stimulation, of SAT (6.25 ± 5.07 vs 5.3 ± 2.08 mL/100 g/min; *P* = .62) and VAT (12.12 ± 6.24 vs 10.45 ± 5.78 mL/100 g/min) were similar between control and obese participants (*P* = .55; Fig. 2).

A mixed meal did not increase SAT blood flow significantly in 20 minutes in obese participants (*P* = .46), but in control individuals there was a significant increase from baseline (*P* < .05) (Fig. 2A). After 50 minutes there was a significant change in SAT blood flow from baseline in obese individuals (*P* < .01) as well as controls (*P* < .05), suggesting a delayed response in obesity (Fig. 2A).

Change of SAT blood flow over time after a mixed meal was blunted in obese participants compared to controls (*P* < .05, interaction time × group). Interestingly, blood flow over time of VAT after the mixed meal was similar between obese and control participants (*P* = .23, interaction time × group; Fig. 2B).

GIP infusion had increased SAT blood flow from baseline after 20 minutes in control (*P* < .01) and obese (*P* < .01) participants before surgery (Fig. 2C). VAT blood flow was also increased 20 minutes after GIP infusion in controls (*P* < .001) and obese individuals (*P* < .001; Fig. 2D). The effects of GIP infusion remained until 50 minutes, and SAT and VAT blood flow were both still increased from baseline in controls (*P* < .001 and *P* < .001 for SAT and VAT, respectively) and in obese participants (*P* < .05 and *P* < .05 for SAT and VAT, respectively).

Even though SAT blood flow was increased by GIP infusion in all groups, change of SAT blood flow over time was blunted in obese individuals compared to controls (*P* < .01, interaction time × group; see Fig. 2C). However, there was only a trend but not a statistically significant difference between obese individuals and controls in VAT blood flow over time in response to GIP infusion (*P* = .051, interaction time × group) (Fig. 2D).

### Changes in Tissue Blood Flow After Bariatric Surgery

After bariatric surgery, obese patients had lost weight (Δ body mass index [BMI] –5.64 ± 1.7, *P* < .01) and insulin sensitivity and glucose homeostasis had improved (HOMA-IR from 5.8 ± 3.4 to 3.1 ± 1.9 fraction; *P* < .01, fasting plasma glucose: 6.53 ± 1.09 to 5.64 ± 0.72 mmol/L; *P* < .05, fasting insulin: 19.4 ± 9.35 to 12.2 ± 7.13 mIU/L; *P* < .05). Bariatric surgery reduced SAT mass by 7.62 ± 3.02 kg, and VAT mass was reduced by 1.56 ± 0.86 kg. SAT and VAT mass both remained higher in obese individuals after bariatric surgery compared to controls (see Table 1).

After bariatric surgery baseline (0 minutes) SAT blood flow was not significantly different between control and obese individuals (6.25 ± 5.07 vs 3.67 ± 1.63 mL/100 g/min; *P* = .17) as was VAT blood flow (12.12 ± 6.24 vs 8.52 ± 3.58 mL/100 g/min; *P* = .16). Likewise, there was no statistically significant change in unstimulated SAT (5.3 ± 2.08 vs 3.67 ± 1.63 mL/100 g/min; *P* = .08) or VAT (10.45 ± 5.78 vs 8.52 ± 3.58 mL/100 g/min; *P* = .16) blood flow after bariatric surgery compared with measurements before surgery.

In obese patients after bariatric surgery, SAT blood flow response to a meal resembled the response of controls (*P* = .46, interaction time × group, after surgery vs controls) (see Fig. 2A). VAT response to a mixed meal over time did not further improve in response to bariatric surgery. SAT blood flow at 20 minutes after a meal increased from baseline in obese participants after surgery (*P* < .01), along with an increase in VAT blood flow (*P* < .01) (Fig. 2B). Similarly, at 50 minutes after a meal SAT (*P* < .05) and VAT (*P* < .01) blood flow were elevated compared to baseline.

After bariatric surgery, GIP infusion increased SAT blood flow from baseline at 20 minutes (*P* < .01) and at 50 minutes (*P* < .05) (see Fig. 2C). After surgery GIP infusion also increased VAT blood flow at 20 minutes (*P* < .01) and at 50 minutes (*P* < .01) (see Fig. 2D) in obese individuals.

SAT blood flow over time after GIP infusion was higher in controls compared with obese patients after surgery (*P* < .05, interaction time × group) (see Fig. 2C). Change of VAT blood flow over time did not differ between controls and obese participants after a mixed meal (*P* = .49, interaction time × group) (see Fig. 2B) or GIP infusion (*P* = .57, interaction time × group) (see Fig. 2D).

There was no difference is SAT or VAT blood between the bariatric surgery groups (Roux-en-Y gastric bypass vs vertical sleeve gastrectomy) after a mixed meal or GIP infusion for time × group interaction.

### Changes in Glucose, Insulin, and Incretin Levels in Response to a Mixed Meal and Glucose-dependent Insulinotropic Peptide Infusion

**Figure 3.**
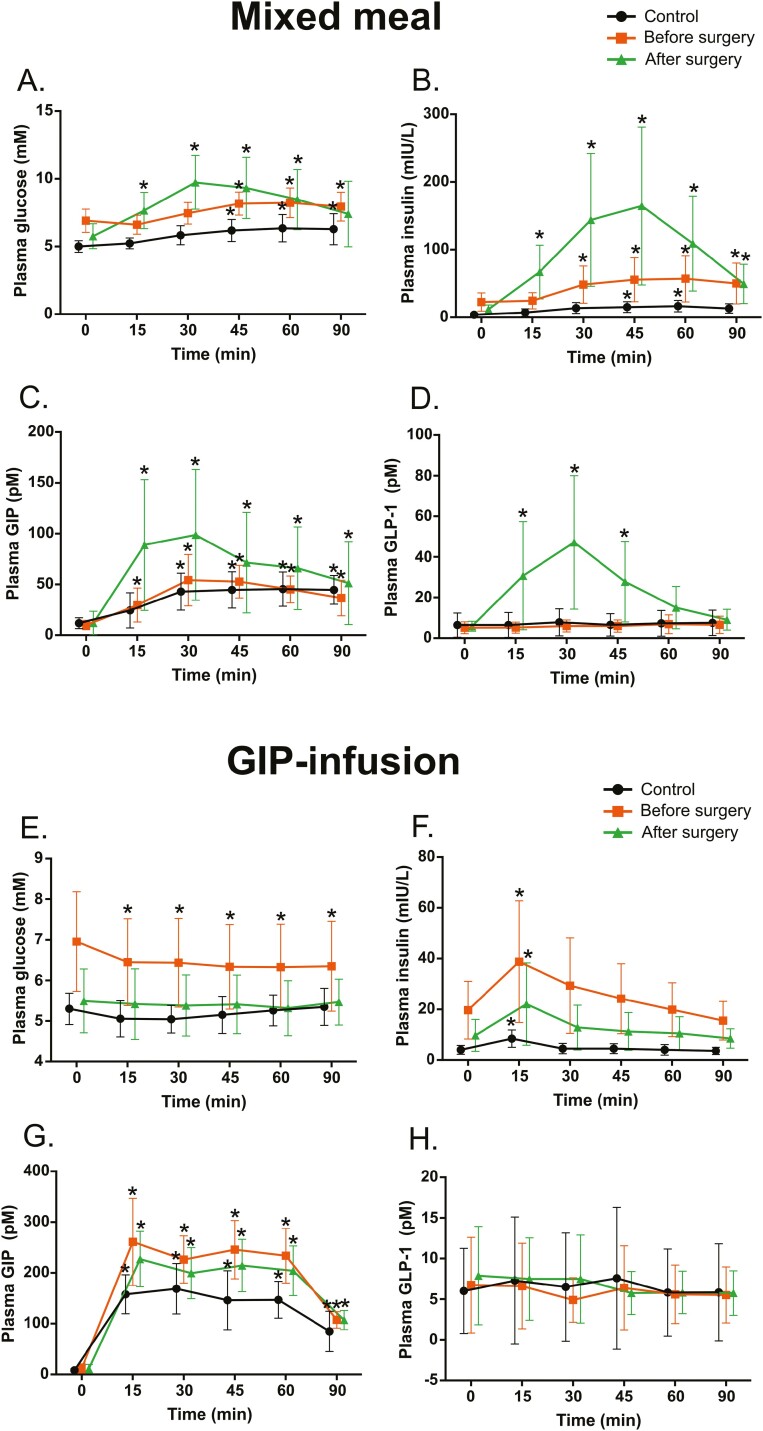
Changes caused by mixed meal stimulation and glucose-dependent insulinotropic peptide (GIP) infusion on plasma glucose, insulin, GIP, and glucagon-like peptide-1 (GLP-1) concentrations in controls and obese individuals before and after bariatric surgery. Data are presented as mean (SD). Asterisk indicates change from baseline (*P* < .05).

We measured changes in plasma glucose, insulin, and incretin (GIP and GLP-1) levels during scanning. These results of glucose, insulin, GIP, and GLP-1 concentrations have been previously reported (25, 26) and are presented in Fig. 3.

After meal stimulation, control and presurgery participants showed an increase of plasma glucose at the 45-minute sample time, whereas postsurgery patients’ more robust plasma glucose response was seen already 15 minutes after the meal (Fig. 3A). Similarly, insulin response after surgery was increased and seen already 15 minutes after the meal (Fig. 3B). Plasma GIP concentrations increased in all individuals after a meal, with a more pronounced increase from baseline in obese patients after surgery (Fig. 3C). Interestingly, we found a significant increase of GLP-1 after a meal in obese individuals after surgery, but not in controls or obese participants before surgery (Fig. 3D).

During GIP infusion plasma glucose levels of obese individuals before surgery decreased from baseline, whereas no change was seen in controls or obese participants after surgery (Fig. 3E). Plasma insulin increased in all groups 15 minutes after the start of GIP infusion (Fig. 3F). As expected, plasma GIP levels increased in all groups (Fig. 3G); however, we did not see significant changes in plasma GLP-1 levels during GIP infusion in any group (Fig. 3H).

Before surgery we found a strong negative correlation during GIP infusion with SAT blood flow area under the curve (AUC) and plasma insulin AUC (rho = –0.83, *P* < .01) and a positive association between SAT blood flow AUC and plasma glucose AUC (rho = 0.73, *P* < .05) during GIP stimulation, as shown in Supplementary Fig. 1 (33). We found no correlations between SAT or VAT blood flow AUC with any of these parameters during mixed-meal testing before bariatric surgery.

After bariatric surgery, the association between plasma insulin AUC and SAT blood flow AUC during GIP infusion were reversed and we found a positive association (rho = 0.905, *P* < .001; see Supplementary Fig. 1) (33). There was also a positive association between SAT blood flow AUC and plasma C-peptide AUC (rho = 0.91, *P* < .01) during GIP infusion. During mixed-meal stimulation a strong association between SAT blood flow AUC and plasma GIP AUC (rho = 0.954, *P* < .001; see Supplementary Fig. 1) was found (33).

In control individuals VAT blood flow AUC correlated with plasma GLP-1 AUC (rho = 0.833, *P* < .01) during mixed-meal testing.

## Discussion

Here we show that meal- and GIP-stimulated SAT blood flow is blunted in obese individuals. Furthermore, the meal-induced SAT blood flow was improved 2 months after bariatric surgery. On the other hand, the response to GIP infusion remained unchanged. The responses to meal and GIP infusion in VAT were similar between lean controls and obese participants before surgery, and no change was seen after bariatric surgery in VAT blood flow under stimulation.

GIP has been shown to increase AT blood flow in the presence of insulin (7), the response being blunted in obesity, possibly due to insulin resistance. We did not find a normalization of AT blood flow after surgery in SAT or VAT during GIP infusion, although insulin sensitivity was indeed enhanced. Weight loss induced by caloric restriction may partially normalize SAT blood flow (8), although variable results exist (34). The study by Viljanen et al (34) used a 6-week very low-calorie diet, and SAT and VAT blood flow both were decreased. The study by Asmar and colleagues (8) used a 12-week weight loss program followed by a 4-week weight-maintenance diet. Participants were leaner than in the present study, and weight loss was not as prominent. However, one may speculate that we would also see a similar result in GIP-induced increase of AT blood flow with a longer follow-up time. At the time of our follow-up measurements, the average weight loss was 17 kg. All participants were still obese and still losing weight at the 2-month follow-up time point. However, we found improved glycemia and 7 out of 10 individuals with T2DM were in remission. Interestingly, we found a strong negative correlation between plasma insulin AUC and SAT blood flow AUC measured during the GIP infusion in obese individuals before surgery, which would indicate a blunted sensitivity to vasostimulatory effects of GIP. Other studies have used the hyperinsulinemic euglycemic clamp technique at the same time as infusion of GIP (8, 12); however, in this study our participants were in a fasting state. Since insulin plays a role in the function of GIP, this might have an effect on the response seen in the fasting state (7). After surgery plasma insulin AUC and SAT blood flow AUC measured during GIP infusion were positively associated, as would be expected. While we saw this change in associations, the increase of SAT blood flow after GIP infusion seen in control participants did not occur in obese participants even after bariatric surgery. It seems that the blunted effects of GIP on SAT blood flow remain after bariatric surgery. In VAT we found an increase in blood flow similar to that in control individuals after GIP infusion, and no further improvement with bariatric surgery.

While the response to GIP infusion remained blunted in SAT, there was a positive relationship between SAT blood flow AUC and plasma GIP AUC during mixed-meal testing after bariatric surgery, suggesting that GIP does have a role in SAT blood flow regulation in the postprandial state. Since GIP infusion did not elicit an increase of SAT blood flow in obese patients after surgery, as it did in control participants, the meal-induced increase of SAT blood flow may be more strongly regulated by some other vasodilators in obese individuals after bariatric surgery, possibly GLP-1. GLP-1 does have vasodilative effects in AT in healthy humans (35). Here we saw an increased GLP-1 release in response to a mixed meal after bariatric surgery, a response much more robust than in control individuals. Besides vasodilative effects, increased secretion of GLP-1 after bariatric surgery has been previously reported and it is possible that it plays a role in reduced food intake after bariatric surgery by affecting appetitive drive and food reward (36, 37). Then again, GLP-1 AUC was associated with VAT blood flow AUC measured after a mixed meal in control participants, but there was no association in the obese group before or after surgery, or associations with SAT blood flow.

VAT is a more metabolically active adipose depot, and it has been more strongly associated with obesity-related complications than SAT (13, 17, 18). Here we found improvement of SAT blood flow in response to a meal, and no change in VAT blood flow over time in response to a meal or a GIP infusion after bariatric surgery. However, the VAT blood flow response to a mixed meal was more rapid after bariatric surgery. It seems that VAT blood flow response to a meal and GIP remains more sensitive in T2DM individuals compared to SAT. The difference between obese and control participants before surgery in VAT blood flow during GIP infusion was not significant, but there was a tendency (*P* = .051). It has been shown that SAT is reduced more by volume after bariatric surgery, but a larger portion of VAT is reduced, and the reductions are mutually associated (19-22). It has been shown that obese individuals’ SAT blood flow is reduced, and SAT blood flow increases after conventional weight loss (34, 38, 39). In a study by Dadson et al (40), VAT was more metabolically active compared to SAT in obese individuals in a fasting state, and VAT adipocyte cell volume was lower compared to SAT. They also reported lower SAT and VAT perfusion values in T2DM and obese participants compared to healthy controls. Here we did not find a significant difference at baseline between controls and T2DM individuals, possibly because of interindividual variation, especially in the control group. However, when perfusion was not expressed as per mass (mL/min/100 g tissue), but rather as per cell number, they did not find a difference between lean and T2DM or obese individuals’ SAT or VAT blood flow. It is possible that the metabolic function of VAT is retained in obesity and T2DM because adipocyte hypertrophy is not as prominent in VAT compared to SAT (40, 41). The improvement in SAT blood flow after bariatric surgery in our present study may not be entirely explained by weight loss; we found no associations between AT depot mass and perfusion, nor did we see any associations between changes in AT mass and perfusion after bariatric surgery. Our follow-up time point at 2 months may be too short to find the ultimate changes both in SAT and VAT depots, and further follow-up time points should be included in future studies. While the follow-up time was short, the results seen here could reflect the effects of rerouting of the gastrointestinal tract more than weight loss. Rerouting the gastrointestinal tract causes nutrients to enter the gut faster, and increased blood flow of the pancreas and intestine improve to adjust for increased nutrient absorption and glucose delivery to and insulin delivery from the pancreatic islets (25, 26, 42). Meal ingestion increases liver, pancreatic, and jejunal blood flow after bariatric surgery, but bariatric surgery does not alter splanchnic or liver blood flow responses to GIP infusion (25, 26). This is in line with our present results, and it seems that GIP by itself does not play a major role in changes seen after bariatric surgery.

A strength of this study is the ability to measure blood flow in SAT and VAT under nonstimulated and stimulated conditions both in lean and obese individuals—as well as before and after bariatric surgery–induced weight loss. However, this study has some limitations. The follow-up time of this study was relatively short: The mixed-meal test was repeated on average 69 days and the GIP infusion test 80 days after bariatric surgery. Since the obese participants were still in the process of rapid weight loss, it is possible that more pronounced changes in AT blood flow could be found later after surgery. We also observed no difference in VAT blood flow between controls and obese individuals, as well as no difference between SAT or VAT blood flow at baseline between controls and obese participants, which could be due to high interindividual variation. Whether the changes are more dependent on changes in insulin sensitivity and glucose homeostasis or weight loss is not obvious. It should be noted that while AT blood flow is linked to glucose uptake and influx and outflux of fatty acids in AT, blood flow does not directly measure the substrate metabolism of AT.

In conclusion, obese individuals with T2DM have a blunted response to mixed-meal ingestion and GIP infusion in SAT. After bariatric surgery SAT blood flow response to a meal improves and resembles the response of healthy controls; however, no improvement was seen in response to GIP infusion. Interestingly these same changes are not seen in VAT: There was no difference between controls and obese participants either after meal ingestion or GIP infusion. After bariatric surgery VAT blood flow increased more rapidly after mixed-meal ingestion, but overall remained the same as before surgery. This emphasizes the higher metabolic activity of VAT, as blood flow remains more sensitive to stimulation by a meal or GIP even in obesity compared with SAT. It seems that the vasodilative effects of GIP are blunted in SAT but not VAT, and that the improved meal response seen in SAT could be mediated via regulators other than GIP.

## Data Availability

Some or all data sets generated during and/or analyzed during the present study are not publicly available but are available from the corresponding author on reasonable request.

## Abbreviations

AT: adipose tissue
AUC: area under the curve
BMI: body mass index
GIP: glucose-dependent insulinotropic peptide
GLP-1: glucagon-like peptide-1
HOMA-IR: homeostatic model assessment of insulin resistance
MRI: magnetic resonance imaging
PET: positron emission tomography
SAT: subcutaneous adipose tissue
T2DM: type 2 diabetes mellitus
TGs: triglycerides
VAT: visceral adipose tissue

## Clinical Trial Information

ClinicalTrials.gov registration number: NCT01880827 (registered June 13, 2013).
